# The Association of Family Incivility with Adolescent Depression: A Moderated Mediation Model

**DOI:** 10.3390/bs14121159

**Published:** 2024-12-03

**Authors:** Miao Miao, Shuai Jin, Yiqun Gan

**Affiliations:** 1 Department of Medical Psychology, School of Health Humanities, Peking University, Beijing 100191, China; miaomiao@bjmu.edu.cn (M.M.); js17801010283@snnu.edu.cn (S.J.); 2 School of Psychological and Cognitive Sciences and Beijing Key Laboratory of Behavior and Mental Health, Peking University, Beijing 100871, China

**Keywords:** family incivility, depression, adolescence, self-compassion, sex differences

## Abstract

Negative family interactions have an adverse impact on adolescent mental health. The present study focused on the influence of family incivility on adolescent depression. In order to examine the association of family incivility with depression, an integrated framework was constructed to explore the mediating role of self-compassion and the moderating role of sex differences. Two waves of data were collected from 999 Chinese senior high school students (43.6% males and 56.4% females), with a mean age of 16.58 ± 0.54 years. Time 1 family incivility was positively associated with depression at Time 2 (*r* = 0.40, *p* < 0.001). Sex differences moderated the link between family incivility and self-compassion, with their association being significant in females (*B* = −0.07, *SE* = 0.03, *p* = 0.013) but not in males (*B* = 0.03, *SE* = 0.03, *p* = 0.376). Regarding the effect of family incivility on depression, the direct effect was significant only in males (*B* = 0.13, *SE* = 0.03, *p* < 0.001), whereas the indirect effect via self-compassion was significant only in females (*B* = 0.01, *SE* = 0.01, 95% CI [0.0010, 0.0232]). The present findings revealed a positive association between family incivility and adolescent depression. Moreover, family incivility has a direct impact on depression in male adolescents and an indirect impact through self-compassion in female adolescents. These findings underscore the important role of adolescent sex differences in the impact of family incivility on adolescent depression and highlight the practical importance of developing interventions to reduce family incivility.

## 1. Introduction

Depression is a prevalent health problem among adolescents [1]. Family environment plays a key role in adolescent development, with a negative family environment (e.g., less support and more conflict) increasing the risk of depression [1,2]. Negative family interactions, such as ignoring children’s opinions and being condescending to them, are regarded as family incivility [3]. While previous research has revealed the impact of family incivility on cyberbullying and interpersonal adaptation among adolescents [4,5,6], its influence on adolescent mental health remains unclear. This study aimed to explore the relationship between family incivility and depression among adolescents. Based on expanded social information processing theory, we explored a mediating mechanism regarding the mediating role of self-compassion between family incivility and depression. Finally, we intended to further identify whether there exist some sex differences regarding the impact of family incivility on depression. An integrated framework was constructed to explore the association between family incivility and depression, with self-compassion as the mediator and sex differences as the moderator.

### 1.1. Influence of Family Incivility on Adolescent Depression

Family factors are robust antecedents of adolescent depression [1,2]. A cohort study found that those who experienced positive family relationships during adolescence had lower levels of depression [1]. One of the most widely examined family factors is family support, which could satisfy adolescents’ needs for belonging, love, and acceptance [7]. Several nationally representative surveys and systematic reviews have revealed the protective role of family support on adolescent mental health such as well-being and depression [2,8]. In addition, numerous studies have revealed the detrimental effect of negative family dynamics on adolescent depression [9,10,11]. In addition to frequently examined negative family interactions such as maltreatment, abuse, and neglect, the present study is focused on family incivility.

Family incivility refers to disrespectful behaviors that violate mutual respect between family members [3], such as ignoring each other and making demeaning comments. Compared with family aggression or abuse, family incivility tends to be subtler and more easily tolerated due to low intensity and ambiguous intentions [3,5]. Nevertheless, family incivility is harmful in the long term. Research among employees showed that retrospectively self-reported family incivility was associated with more psychological distress [3] and family–work conflicts [12]. In addition, family incivility was positively linked with hopelessness [4], negative affect [5], loneliness, and interpersonal adaption [6] among adolescents and young adults. According to Apsley and Padilla-Walker [13], when parents engage in incivility behavior such as criticism, neglect, and belittlement, adolescents will model these behaviors and become more critical and rigorous toward themselves, thus increasing the risk of depression. However, few studies have examined the influence of family incivility on adolescent psychopathology, particularly in relation to depression. By combining previous evidence on the impact of family factors on depression with empirical findings on the relationship between family incivility and mental health, we expect family incivility to be a risk factor for adolescent depression.

### 1.2. Self-Compassion as a Mediator

Self-compassion represents a positive self-construct and refers to being understanding and kind to oneself, especially when faced with personal failure or suffering. Self-compassion comprises three basic components: self-kindness, common humanity, and mindfulness [14]. Self-kindness is defined as responding to personal sufferings such as personal inadequacy or life difficulties in a supportive and kind way; common humanity refers to a cognitive understanding of these personal sufferings and treating them as part of common human experiences; and mindfulness represents a balanced awareness of these personal sufferings, i.e., neither exaggerating nor avoiding personal pains or negative feelings [15]. Based on a frequently used six-dimension assessment for self-compassion—the Self-Compassion Scale [16], both these three self-compassionate self-responding components and their counterparts—unself-compassionate components (self-judgment, isolation, and over-identification)—were included in the assessment of self-compassion.

A systematic review has found that self-compassion is negatively associated with adolescent psychopathology [17]. Specifically, self-compassion has proved to be an effective emotion regulation strategy for depression [18], and empirical research has revealed a negative predictive role of self-compassion on depression [19]. Moreover, self-compassion may play an important role in the relationship between family factors and mental health, with higher self-compassion associated with higher family support and less depressive symptoms [20]. Neff and McGehee [21] further found that self-compassion partially mediated the relationship between family factors (e.g., maternal support and family functioning) and well-being. Similarly, Jin, Liu and Miao [6] found that self-compassion mediated the impact of family incivility on interpersonal adaptation and loneliness. Based on social information processing theory, this study aimed to investigate the mediating role of self-compassion between family incivility and adolescent depression.

Social information processing theory posits that cues in the social environment activate different information processing processes that shape children’s cognition, affect, and behavior [22]. Furthermore, Lau and Waters [23] proposed an expanded version of this theory for adolescent anxiety and depression, suggesting that depression and anxiety emerge from the interplay of distal risk factors (e.g., genetics and environmental experiences) and proximal information-processing mechanisms (e.g., appraisal biases). Specifically, information-processing factors mediate the impact of distal risk factors on clinical symptoms. According to Lau and Waters [23], dysfunctional family dynamics, such as abuse and neglect, act as typical environmental risk factors. Information-processing variables, especially attention biases and appraisal biases, act as candidate mediators between distal risk factors and adolescent depression. Therefore, it may be that family incivility, acting as distal risk factor, triggers negative information processing such as attention and appraisal biases toward oneself, which then manifests as less compassionate self-responding and in turn causes depression.

### 1.3. Moderating Role of Adolescent Sex Differences

Considering that adolescence is a crucial developmental period and that male and female adolescents differ in their social functioning and reactions to stress [24], the present study aimed to further examine the possible sex differences in the impact of family incivility on adolescent depression. Compared to male adolescents, female adolescents are at higher risk of depression [1,25]. Moreover, according to Chen and Harris [1], compared with males, females are more likely to benefit from positive family interactions in reducing depression. However, more research is needed to clarify whether the impact of negative family interaction on adolescent depression differs between the sexes.

Empirical evidence on the sex difference in the level of experienced family incivility is inconsistent. For instance, Jin, Liu and Miao [6] found non-significant differences between male and female high school students. However, Bai, Bai, Huang, Hsueh and Wang [4] found in another adolescent sample that boys experienced higher levels of family incivility than girls, which was consistent with Jin and Miao’s [5] findings among university students. Therefore, more research is needed to explore whether adolescent males and females experience different levels of family incivility. 

Moreover, the relationship between the sex difference and the impact of family incivility on adolescent mental health remains unknown. A systematic review pointed out that experiencing stress during puberty and early adulthood has a stronger proximal impact on females than males because females are more sensitive and vulnerable to stress [26]. When encountering stress such as maltreatment, girls are more reactive to stress and more likely to adopt internalizing coping strategies such as rumination and self-criticism [27], thereby increasing the risk of developing internalizing disorders such as depression [28]. For instance, female adolescents reported higher levels of interpersonal stress than male adolescents and showed greater cortisol reactivity to interpersonal challenges, such as social rejection [29]. In addition, females are more reactive to emotional stimuli, especially unpleasant or threatening ones [30]. Considering that family incivility could trigger negative affect [4,5], it is reasonable to suppose a severer impact of family incivility on mental health among female adolescents than male adolescents.

As for the hypothesized mediating role of self-compassion, a previous review has revealed that males demonstrate higher levels of self-compassion than females [31]. Similar results were also obtained in various adolescent samples [32,33,34]. Due to hormonal changes during adolescence, females are more vulnerable to stress and adverse experiences [26], thus leading to poor hypothalamic–pituitary–adrenal axis functioning and higher cortisol activity [35]. In addition, females are more likely to overreact to emotional information, thus increasing the risk of disorder in emotion regulation [36]. Therefore, when encountering adverse events such as family incivility, female adolescents tend to appraise these experiences in a more negative way; hence, they are more likely to engage in biased information processing, manifesting as less self-compassionate self-responding. Hence, we suppose that the sex difference moderates the indirect effect of family incivility on depression via self-compassion, with the indirect effect being stronger on females than males.

### 1.4. The Present Study

**Figure 1:**
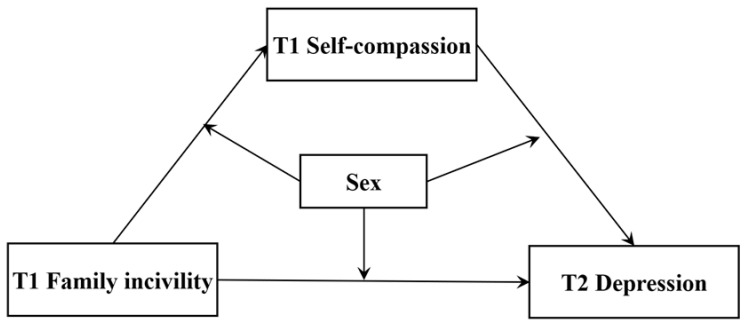
Hypothesized moderated mediation model. Note: Depression at T1 was included as a covariate to control for baseline depression levels, ensuring that the observed effects of family incivility on depression at T2 are not confounded by pre-existing differences in depression.

This study aimed to examine the relationship between family incivility and adolescent depression. Building on social information processing theory, we proposed a conceptual model in Figure 1 to explain the underlying mechanism for the impact of family incivility on depression. Moreover, given the sex differences in response to stress and adverse events during adolescence and the differing impact of family interactions on adolescent depression, the present study aimed to further investigate sex as a moderator on the impact of family incivility on depression. Specifically, we proposed the following hypotheses:

**Hypothesis 1** **(H1).** *family incivility is positively associated with depression.*

**Hypothesis 2** **(H2).** *self-compassion mediates the relationship between family incivility and depression.*

**Hypothesis 3** **(H3).** *sex differences moderate the relationship between family incivility and depression such that their association is stronger in female adolescents than male adolescents.*

**Hypothesis 4** **(H4).** *sex differences moderate the indirect effect of family incivility on depression via self-compassion, with the indirect effect being stronger on female adolescents than male adolescents.*

## 2. Method

### 2.1. Participants and Procedure

A two-wave longitudinal design was employed to examine the mediating role of self-compassion between family incivility at Time 1 (T1) and depression at Time 2 (T2). Specifically, depression was assessed at both time points to account for its baseline level. For the hypothesized mediating model, the Monte Carlo Power Analysis for Indirect Effects (https://schoemanna.shinyapps.io/mc_power_med/) (accessed on 27 November 2024) written by Alexander M. Schoemann, Aaron J. Boulton, and Stephen D. Short was used to estimate the sample size. Based on the results by Jin et al. (2023), we set the correlation between family incivility and self-compassion at 0.3, and according to Marsh, Chan and MacBeth [17], we set the correlation between self-compassion and depression at 0.52. For the correlation between family incivility and depression, we used a moderate value of 0.3. The power analysis indicated that to obtain a minimum power of 0.8, at least 90 participants were needed. Of note is that we were unable to evaluate the minimum sample size for the hypothesized moderated mediational model, since characterizing effect sizes in such models are not clearly understood. However, we tried to recruit a large enough sample for the model analyses.

Participants were recruited through convenience sampling in a senior high school in a county in Guangdong Province, China. The questionnaires were administered in Chinese to all twenty-four classes in the senior-two grade. Two waves of data were collected one month apart. Questionnaires that displayed response patterns and failed on the infrequency/attention check item were considered invalid data. In total, 1079 and 1050 adolescents provided valid data at T1 and T2, respectively. Data from adolescents at different time points were matched through the participants’ unique ID, which was generated for and in this study. Finally, data from 999 adolescents (56.4% female and 43.6% male) were matched and included in the final analyses, with their age ranging from 15 to 19 years (mean [M] = 16.58 ± 0.54). Among them, results showed that 177 (17.7%) were only-child adolescents, 821 (82.2%) had at least one sibling, and one did not report this information. Regarding parental education, since the data were collected in a remote county, 78.1% of mothers and 71.9% of fathers had an education level below senior high school. See Table 1 for detailed demographic information.

This study was approved by the ethics committee of the corresponding author’s institution. Before data collection, approval for this study was obtained from the school administrator, class teachers, and adolescents’ parents. All adolescents voluntarily participated in this study, and informed consent was obtained from them prior to data collection. Each survey was group tested using paper and pencils. To generate the unique IDs used to match the two waves of data, participants were required to write down their birthdays and the last four digits of their phone numbers in each survey. The participants completed the questionnaires in quiet classrooms and were given small gifts (a bookmark or a key chain) upon completion of each survey. 

### 2.2. Measures

Family incivility was assessed at T1 using the 6-item Family Incivility Scale [3], which measures the frequency of uncivil behaviors experienced in the family. Sample items included “Put you down or was condescending to you” and “Ignored or excluded you from social activities.” Responses were given on a 5-point Likert-type scale ranging from 1 to 5 (1 = not at all, 5 = most of the time). The Chinese version showed satisfactory reliability among Chinese adolescents [4]. A previous study among Chinese adolescents further tested the scale’s reliability and validity. The results of confirmatory factor analysis yielded acceptable results, and both Cronbach’s alpha and McDonald’s Omega were satisfactory, reaching 0.92 [6]. In the current study, the Cronbach’s alpha was 0.84.

Self-compassion was measured at T1 using the Chinese version of the Self-Compassion Scale [16], which comprises six subscales, namely self-kindness, common humanity, mindfulness, self-judgment, isolation, and over-identification. Responses were given on a 5-point Likert scale ranging from 1 to 5 (1 = not at all true of me, 5 = very true of me). In the present study, the Cronbach’s alphas ranged from 0.59 to 0.79 for the six subscales and reached 0.88 for the total scale.

Depression was assessed at both time points with the Chinese version of the Depression Anxiety Stress Scale [37,38], which comprises three 7-item subscales: depression, anxiety, and stress. Only the depression subscale was used in the present study. Responses were given on a 4-point Likert scale ranging from 1 to 4 (1 = not applicable to me, 4 = applicable to me often or most of the time). The Cronbach’s alpha was 0.82 at T1 and 0.87 at T2.

### 2.3. Data Analysis

SPSS version 25 was used to conduct attrition analyses, descriptive analyses, and Pearson’s correlations. Then, the PROCESS macro in SPSS was used to conduct moderated mediation analyses [39]. Model 59 was used, with bootstrap samples set at 5000. The index of moderated mediation (IMM) was also calculated to examine whether the hypothesized mediation pathway was significantly moderated by sex differences [39].

## 3. Results

### 3.1. Attrition Analyses

Regarding the final sample (N = 999) and those who dropped out (N = 80), no significant differences were found in age, the father’s education level, whether an only child or not, and the study variables. Nevertheless, compared with the final sample (436 males and 563 females), those who dropped out (47 males and 33 females) were more likely to be male (*χ*^2^ = 6.84, *p* = 0.009). In addition, the mother’s education level was higher among those who dropped out (M = 2.61 ± 1.20) than the final sample (M = 2.18 ± 0.96, t = 3.09, *p* = 0.003, Cohen’s d = 0.44).

### 3.2. Descriptive Statistics

Table 2 displays the means, standard deviations (SD), and correlations among study variables. Results showed that family incivility showed a negative association with self-compassion (*r* = −0.27, *p* < 0.001) and a positive association with depression (*r*_*T*1_ = 0.42, *p* < 0.001; *r*_*T*2_ = 0.40, *p* < 0.001). Therefore, H1 was supported. Regarding demographics, both sex and the only-child status demonstrated a low level of association with self-compassion, with females and those who have siblings demonstrating a slightly low level of self-compassion. In addition, the results of analysis of variance (ANOVA) revealed no significant differences between groups based on the father’s and mother’s educational levels for any of the study variables (*p*s > 0.05), including family incivility, self-compassion, and depression. Therefore, only the demographics of the only-child status was controlled in the subsequent analyses.

### 3.3. Moderated Mediation Analyses

**Figure 2:**
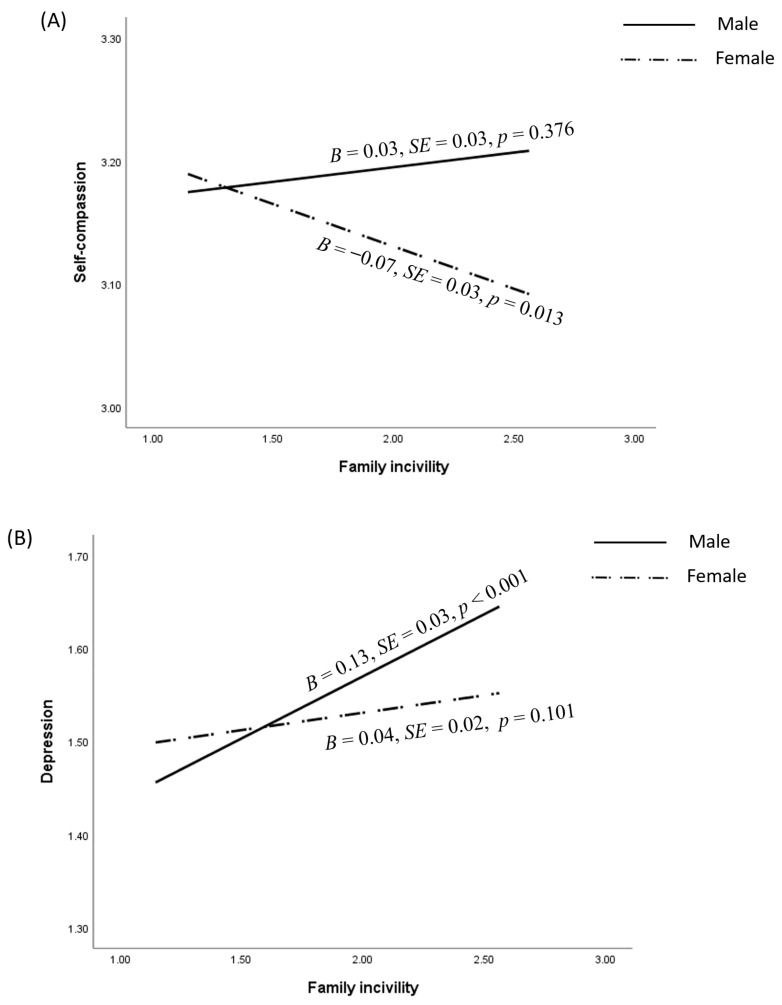
Moderating role of sex differences on the impact of family incivility: (**A**): indicates the simple slope analyses of family incivility on self-compasission; (**B**) indicates the simple slope analyses of family incivility on depression.

As is shown in Table 3, the results revealed a significant moderating role of sex on the association between family incivility and self-compassion: *B* = −0.09, *SE* = 0.04, *p* = 0.015. Specifically, as can be seen in Table 4, family incivility was negatively linked to self-compassion among female adolescents, *B* = −0.07, *SE* = 0.03, *p* = 0.013, whereas their association was non-significant among male adolescents, *B* = 0.03, *SE* = 0.03, *p* = 0.376 (Figure 2A). In addition, sex also moderated the direct association between T1 family incivility and T2 depression, *B* = −0.10, *SE* = 0.03, *p* = 0.004. Specifically, the results in Table 4 showed that the direct association between family incivility and depression was significant among male adolescents, *B* = 0.13, *SE* = 0.03, *p* < 0.001, while this effect was non-significant among female adolescents, *B* = 0.04, *SE* = 0.02, *p* = 0.101 (Figure 2B). Therefore, H3 was partly supported. However, sex did not moderate the negative association between T1 self-compassion and T2 depression.

Furthermore, as is shown in Table 4, sex moderated the indirect effect of family incivility on depression via self-compassion, IMM = 0.01, *SE* = 0.01, 95% [0.0010, 0.0296]. Specifically, the indirect effect was significant only in female adolescents (*B* = 0.01, *SE* = 0.01, 95% [0.0010, 0.0232]) and not in male adolescents (*B* = −0.003, *SE* = 0.01, 95% [−0.0137, 0.0055]). Therefore, H2 was supported among female adolescents, and H4 was supported.

Additionally, due to the short interval between T1 and T2, the strong autoregressive effect of depression may have reduced the coefficients of other variables on T2 depression in the model. To address this, we re-ran the moderated mediation analyses without controlling for T1 depression. The results remained consistent, with sex significantly moderating the mediating pathway from family incivility to depression via self-compassion. Detailed results are provided in the Tables S1 and S2. 

## 4. Discussion

Family-related environmental factors have significant impact on adolescent mental health [1,40]. Regarding negative family interaction, the previous research was mainly focused on severe factors such as family aggression or abuse. However, the present study was concerned with a more subtle but common interaction—family incivility. Drawing on expanded social information processing theory [23], this study proposed the underlying mechanisms for the effect of family interaction on adolescent depression: family incivility impairs self-compassion, which further leads to depression. The results showed that the impact of family incivility varied by sex. For male adolescents, family incivility demonstrated a significant direct effect on depression, while for female adolescents, family incivility was associated with depression indirectly via self-compassion. These findings suggest that family incivility places adolescents at a high risk of developing mental health problems, such as depression, in both the male and female adolescents. In addition, it brings researchers’ attention to sex differences and highlights the importance of considering these differences when developing interventions of family incivility for adolescents.

### 4.1. Indirect Association of Family Incivility with Depression via Self-Compassion Among Females

While previous research has revealed a close association between family factors and adolescent depression, empirical research on family incivility remains limited, despite such incivility being common in parent–adolescent interactions, often ignored by those affected by it, and having the potential to cause enduring impacts [4,5]. The present results found that T1 family incivility was positively associated with depression at both T1 and T2. Further analyses found that this association varied by sex. For female adolescents, the direct effect of T1 family incivility on T2 depression was non-significant when controlling for T1 depression. On the contrary, family incivility demonstrated an indirect effect on depression via self-compassion, which is in line with Jin and Miao’s [6] findings that self-compassion mediated the relationship between family incivility and mental health (i.e., loneliness and interpersonal adaption).

From a social development perspective, social relationships play a key role in developing a positive and healthy view of the self in adolescents [22,41]. Specifically, the view of the self (e.g., self-compassion and self-esteem) is mainly based on social information, such as social relationships and feedback from social connections [41]. Hence, family incivility would destroy positive parent–adolescent relationships and generate negative feedback, thus impacting the development of a healthy view of the self, such as self-compassion. Furthermore, based on social information processing theory [23], the present findings supported the hypothesis that self-compassion acts as a proximal information-processing mechanism between distal risk factors of family incivility and adolescent depression. Dysfunctional family interactions will trigger negative view of the self, thus leading to a critical self-view, which is more likely to cause mental health problems. In contrast, less incivility and more support can foster positive and compassionate self-views because compassion and care are modeled by parents in supportive relationships [21]. When faced with personal sufferings, these adolescents are more likely to internalize a positive self-view, thus alleviating mental health problems.

### 4.2. Direct Association of Family Incivility with Depression Among Males

For male adolescents, the indirect effect of family incivility on depression was non-significant; instead, it showed a direct effect on depression. Specifically, contrary to the results among females, the association between family incivility and self-compassion was non-significant for males. Compared to male adolescents, female adolescents are more vulnerable to stress and show an increased risk of developing affective disorders such as anxiety and depression [42,43]. Due to the influence of sex hormones [42,43], especially during puberty, females are more likely to adopt emotional coping strategies or internalizing coping strategies such as self-criticism when encountering stress and adverse experiences, whereas males tend to engage in problem-solving strategies instead of emotional ones [27]. Moreover, females tend to perceive life stress and maltreatment as more negative, serious, and uncontrollable than males [36]. Therefore, when parents interact with their children in an uncivil way, for instance, belittling or doubting their judgment and neglecting or excluding them, it can be particularly harmful to the emotion regulation of female adolescents. Hence, consistent with the previous empirical studies that found sex differences moderated the association of emotional abuse and neglect with difficulty in identifying feelings (with stronger impacts in females) [36], the present study found that the negative association of family incivility with self-compassion was also stronger in female adolescents than male adolescents.

However, contrary to our hypothesis that the association between family incivility and depression is higher in females than males (H3), the direct association between them was significant only in males but not females. These findings suggest that family incivility might also do harm to male adolescents’ mental health and highlighted the need to further examine the underlying mechanism for them. A previous study found that parental neglect demonstrated a stronger association with hope in boys than girls [44]. Given that hope is a vulnerability factor for depression and that various research has revealed the predictive role of hope on depression [45], future research could examine whether hope could explain the impact of family incivility on depression among male adolescents. Additionally, it should be noted sex differences in response to stress and negative events and the related emotional responses might only exist during periods of significant hormonal fluctuation such as puberty, pregnancy, and menopause [26,46]; caution should be exercised when generalizing the present findings on sex differences to other groups.

### 4.3. Implications

This study was focused on the association between family incivility and depression, with sex differences taken into consideration. The present findings revealed that family incivility is a vulnerability factor for depression in both male and female adolescents, with the underlying mechanism varies with sex. Theoretically, our evidence conforms to previous findings and adds to the literature in three ways. First, previous studies on family risk factors for adolescent depression have mostly focused on childhood trauma (e.g., bereavement) and dysfunctional family interaction (e.g., abuse) and overlooked mild stressors, such as family incivility. The present findings highlight the negative impact of family incivility on adolescent depression. Second, this study extends social information processing theory to the field of family incivility and promotes our understanding of the mechanisms underlying the influence of family interaction on depression. Third, the present study contributes to the literature by revealing sex differences in response to family incivility, which is directly associated with depression for male adolescents but indirectly associated with depression via self-compassion for female adolescents. These findings can deepen the understanding of the psychopathological differences in the impact of negative family interaction or maltreatment between the sexes.

The present findings have practical implications for the promotion of adolescent mental health. First, it underpins the adverse effects of family incivility on adolescent depression. Family incivility is easily ignored and difficult to restrain [4], making it a risk factor for the struggles in obtaining a healthy view of the self and developing mental health problems in the long run. Therefore, parents should be educated regarding the influence of family incivility and improve their awareness and self-reflection about uncivil behaviors in parent–adolescent interactions. Interventions to promote mutual respect and strengthen supportive parent–adolescent interactions are needed. Tara, Turpyn, Fischer, Martelli, Ross, Leichtweis, Miller and Sinha [47] found that parenting-focused mindfulness interventions could increase mindful parenting and promote parent–adolescent relationships, so it might be a useful design when attempting to increase parents’ mindfulness regarding their uncivil behaviors toward adolescents.

Second, the moderating role of sex differences on the direct and indirect association between family incivility and depression highlighted the importance of considering sex differences while developing interventions for the prevention of family incivility for adolescents. Given the indirect association of family incivility with depression via self-compassion among female adolescents, in addition to parenting-focused interventions, developing self-compassion interventions might help to block the negative impact of family incivility on mental health.

### 4.4. Limitations and Future Directions

Some limitations of this study should be noted. First, although a two-wave design was used, the relatively short one-month interval between T1 and T2 limited the examination of long-term associations between study variables. Moreover, when controlling for baseline depression, the strong autoregressive effect likely resulted in smaller regression coefficients for other variables. Therefore, future research should collect at least three waves of data over a longer time period to assess the longitudinal relationships between family incivility, self-compassion, and depression and to better capture the influence of family incivility on changes in depression over time. Second, due to the observational nature of the study design and the use of self-report assessments, we should be cautious when drawing causal conclusions. Future researchers could adopt experimental and interventional designs to replicate the present findings. Additionally, while family incivility is usually assessed by self-report tools, such an approach makes excluding the possibility of self-report bias a difficult undertaking. The uniformity of this construct also limits the exploration of diverse forms of family incivility. Hence, scholars are encouraged to consider objective or observer-reported assessments, such as parent–child interaction observations, to address these limitations. Third, considering that participants were recruited from the same grade of a single senior high school in China, their demographics were homogeneous. Moreover, given that the majority of the adolescents’ parents’ education levels were below senior high school, the present sample had a relatively low socioeconomic status. More diverse samples are needed to generalize the present findings to groups of different ages, socioeconomic status levels, and cultures. Finally, given that paternal and maternal attachment has different impacts on adolescents’ self-compassion and self-evaluation between the sexes [48,49], more research is needed to examine whether the impact of family incivility varies between the parent and adolescent depending on their respective sex.

## 5. Conclusions

The present study revealed a positive link between family incivility and adolescent depression and sex differences were observed. Specifically, the direct association between family incivility and depression was significant in males, but non-significant in females. On the contrary, the indirect effect of family incivility on depression via self-compassion was significant in females, but not in males. When developing interventions for the prevention of family incivility, sex differences should be taken into consideration.

## Figures and Tables

**Table 1:** Demographic information of the sample.

	Cases	Percentage (%)
Sex		
Female	563	56.4
Male	436	43.6
Only-child		
Yes	177	17.7
No	821	82.2
Missing value	1	0.1
Father’s education		
Primary school or below	105	10.5
Junior high school	613	61.4
Senior high school	149	14.9
college	63	6.3
Bachelor or above	62	6.2
Missing value	7	0.7
Mother’s education		
Primary school or below	184	18.4
Junior high school	596	59.7
Senior high school	99	9.9
college	65	6.5
Bachelor or above	44	4.4
Missing value	11	1.1

**Table 2:** Means, standard deviations, and correlations among study variables (N = 999).

	M	SD	1	2	3	4	5	6
1 Age	16.58	0.54						
2 Sex	-	-	−0.08 *					
3 Only child	-	-	0.02	0.16 **				
4 T1 family incivility	1.85	0.71	0.01	−0.02	−0.03			
5 T1 self-compassion	3.16	0.52	−0.02	−0.08 *	−0.07 *	−0.27 **		
6 T1 Depression	1.56	0.50	−0.01	0.03	0.00	0.42 **	−0.57 **	
7 T2 Depression	1.54	0.54	0.04	0.004	0.01	0.40 **	−0.51 **	0.74 **

Note: Both sex and the only-child status were binary coded (Sex: 1 = male, 2 = female; Only child: 1 = yes, 2 = no). * *p* < 0.05. ** *p* < 0.01.

**Table 3:** Unstandardized regression results of the moderated mediation model.

	T1 Self-Compassion			T2 Depression		
	*B*	*SE*	*p*	*B*	*SE*	*p*
Only child	−0.09	0.04	0.018	0.02	0.03	0.594
T1 Depression	−0.58	0.03	<0.001	0.66	0.03	<0.001
X (T1 FI)	0.12	0.06	0.059	0.23	0.05	<0.001
M (T1 self-compassion)				−0.07	0.08	0.357
W (Sex)	0.12	0.08	0.121	0.29	0.17	0.089
X × W	−0.09	0.04	0.015	−0.10	0.03	0.004
M × W				−0.04	0.05	0.327

**Table 4:** The results of conditional effects.

Conditional Effects	*B*	*SE*	*p*	Boot LLCI	Boot ULCI
Direct effect of family incivility on self-compassion					
Male	0.03	0.03	0.376	−0.0326	0.0861
Female	−0.07	0.03	0.013	−0.1206	−0.0140
Direct effect of family incivility on depression					
Male	0.13	0.03	<0.001	0.0810	0.1837
Female	0.04	0.02	0.101	−0.0074	0.0827
Indirect effect of family incivility on depression via self-compassion					
Male	−0.003	0.01		−0.0137	0.0055
Female	0.01	0.01		0.0010	0.0232
Index of moderated mediation	0.01	0.01		0.0010	0.0296

## Data Availability

The raw data supporting the conclusions of this article will be made available by the authors upon request.

## Supplementary Materials

The following supporting information can be downloaded at: https://www.mdpi.com/article/10.3390/bs14121159/s1, Table S1: Unstandardized regression results of the moderated mediation model without controlling for T1 depression; Table S2: The results of conditional effects without controlling for T1 depression.
