# Supplementary material for: The Association of Family Incivility with Adolescent Depression: A Moderated Mediation Model
Source: Behav Sci (Basel). 2024 Dec 3;14(12):1159. doi: 10.3390/bs14121159 (PMC11673280; doi:10.3390/bs14121159)
Supplement: Supplementary file 1 [file behavsci-14-01159-s001.zip › behavsci-3291796-supplementary.pdf]

Supplemental materials

Table S1. Unstandardized regression results of the moderated mediation model without controlling for T1 depression

	T1 self-compassion			T2 Depression		
	B	*SE*	*p*	*B*	*SE*	*p*
Onlychild	-0.09	0.04	0.031	0.01	0.04	0.892
X (T1 FI)	-0.07	0.07	0.372	0.43	0.07	<0.001
M (T1 self-compassion)				-0.34	0.09	<0.001
W (Sex)	0.08	0.09	0.354	0.46	0.21	0.029
X × W	-0.09	0.05	0.059	-0.14	0.04	<0.001
M × W				-0.07	0.06	0.187

Table S2. The results of conditional effects without controlling for T1 depression

Conditional effects	*B*	SE	*p*	Boot LLCI	Boot ULCI
Direct effect of family incivility on self-compassion					
Male	-0.15	0.03	<0.001	-0.2180	-0.0854
Female	-0.24	0.03	<0.001	-0.2965	-0.1782
Direct effect of family incivility on depression					
Male	0.29	0.03	<0.001	0.2334	0.3514
Female	0.15	0.03	<0.001	0.1004	0.2085
Indirect effect of family incivility on depression via self-compassion					
Male	0.06	0.02		0.0314	0.0974
Female	0.11	0.02		0.0818	0.1507
Index of moderated mediation	0.05	0.02		0.0038	0.1014
